# Formulation and Characterization of Aceclofenac-Loaded Nanofiber Based Orally Dissolving Webs

**DOI:** 10.3390/pharmaceutics11080417

**Published:** 2019-08-17

**Authors:** Emese Sipos, Nóra Kósa, Adrienn Kazsoki, Zoltán-István Szabó, Romána Zelkó

**Affiliations:** 1 Department of Drugs Industry and Pharmaceutical Management, University of Medicine, Pharmacy, Sciences and Technology of Targu Mures, Gheorghe Marinescu 38, 540139 Targu Mures, Romania; 2 University Pharmacy Department of Pharmacy Administration, Semmelweis University, H-1092 Hőgyes Endre utca 7-9, 1092 Budapest, Hungary

**Keywords:** aceclofenac, nanofiber, electrospinning, scanning electron microscopy, fourier transform infrared spectroscopy, differential scanning calorimetry

## Abstract

Aceclofenac-loaded poly(vinyl-pyrrolidone)-based nanofiber formulations were prepared by electrospinning to obtain drug-loaded orally disintegrating webs to enhance the solubility and dissolution rate of the poorly soluble anti-inflammatory active that belongs to the BCS Class-II. Triethanolamine-containing ternary composite of aceclofenac-poly(vinyl-pyrrolidone) nanofibers were formulated to exert the synergistic effect on the drug-dissolution improvement. The composition and the electrospinning parameters were changed to select the fibrous sample of optimum fiber characteristics. To determine the morphology of the nanofibers, scanning electron microscopy was used. Fourier transform infrared spectroscopy (FT-IR), and differential scanning calorimetry (DSC) were applied for the solid-state characterization of the samples, while the drug release profile was followed by the in vitro dissolution test. The nanofibrous formulations had diameters in the range of few hundred nanometers. FT-IR spectra and DSC thermograms indicated the amorphization of aceclofenac, which resulted in a rapid release of the active substance. The characteristics of the selected ternary fiber composition (10 mg/g aceclofenac, 1% *w*/*w* triethanolamine, 15% *w*/*w* PVPK90) were found to be suitable for obtaining orally dissolving webs of fast dissolution and potential oral absorption.

## 1. Introduction

Nonsteroidal anti-inflammatory drugs (NSAIDs) are often used in the therapy of osteoarthritis and rheumatoid arthritis for pain relief and to reduce inflammation. NSAIDs inhibit both isoforms of cyclooxygenase enzyme (COX), but their analgesic and anti-inflammatory effects are due to inhibition of COX-2 [1]. The adverse effects such as gastrointestinal bleeding and ulcer, stroke, heart attacks are primarily caused by the inhibition of COX-1 [2,3]. The risk of side effects increases with high doses and long duration of systemic exposure to NSAIDs [4].

Aceclofenac is a potent NSAID with significant anti-inflammatory and analgesic properties. Its effect is achieved by inhibiting the production of inflammatory mediators including prostaglandin E2 (PGE2), tumor necrosis factors (TNF) and different interleukins (IL1-β, IL-2) [5,6]. Aceclofenac also has chondroprotective effects by the synthesis of glycosaminoglycan [6,7,8]. It is reported to be more potent or at least to have similar anti-inflammatory effects than conventional NSAIDs in double-bind studies [9,10]. Aceclofenac has a better gastric tolerance, explained by its higher selectivity towards COX-2 than COX-1, which explains its safety compared to traditional NSAIDs and COX-2 selective inhibitors [1,6,8]. The drug is practically insoluble in water, belongs to the Biopharmaceutical Classification System (BCS) Class II. [6] After oral administration aceclofenac is well absorbed, however, its bioavailability is reduced due to high first pass metabolism and poor aqueous solubility of the drug [6,8].

There are numerous technological methods to increase the bioavailability of a drug. For BCS class II drugs, this can be achieved by enhancing the dissolution of the poorly water-soluble active substance by reducing particle size, preparation of water-soluble complexes, using surfactant systems, liposomes, and formulation of solid dispersions [11]. Among the different kinds of solid dispersion strategies new type of solid dispersions, typically ternary or quaternary systems, with one or two additives (such as surfactants and other polymeric excipients) that, together with the drug and host polymer, are combined to exert a synergistic effect on drug-dissolution improvement. The polymer-based solid dispersions are amorphous composites [12].

The oral bioavailability of aceclofenac could be enhanced using chitosan cocrystals trapped into the alginate matrix as a supersaturated drug delivery system (SDDS) or using polymeric microspheres, which can act as delivery systems for sustained drug release [13,14,15,16]. The production of nanocrystals using the surface solid dispersion technique is also an appropriate method to improve the dissolution rate of aceclofenac [17,18]. Triethanolamine was also used to prepare aceclofenac salt to improve the solubility [19]. In order to minimize the gastrointestinal side effects and to attain controlled release delivery of the drug, the development of starch-blended Ca^2+^-Zn^2+^-alginate microparticles and aceclofenac-loaded interpenetrating network (IPN) nanocomposites were also described [20,21].

Nowadays nanotechnology is an evolving area for the development of drug delivery systems with dimensions between 1–100 nm [22]. Nano-carriers have high surface area to volume ratio, can reduce the side effects of conventional pharmaceuticals and they are successful carriers for insoluble active ingredients [23]. Core/shell nanoscale particles were prepared with a modified coaxial electrospraying process in which a very thin shell layer was employed to give very rapid dissolution of a poorly water soluble helicid [24]. Nanofiber-based drug delivery systems own their high drug uptake due to their large surface area and porosity, but they are also able to simultaneously deliver more than one drug or can be used as transdermal drug delivery systems, as well [25].

These nanofibers can be successfully used to prepare orally dissolving webs, an innovative group of drug delivery systems with several advantages over existing oral formulations such as:

- rapid disintegration and high dissolution rate in the oral cavity due to the large surface area,
- no need of water for administration; this could be an advantage in geriatrics, pediatrics, but also for dysphagic patients or patients who are unable to swallow tablets and capsules or a large amount of water,
- precise dosage administration in each film,
- rapid absorption from the highly vascularized buccal mucosa—drugs are absorbed directly to the superior vena cava, entering into the systemic circulation without pre-systemic metabolism,
- increase in bioavailability and rapid onset of action by avoiding the first-pass hepatic metabolism, while the dose can be reduced, which can lead to a reduction of side effects [26,27].

The nanofibrous formulations enable sustained drug release, as well. Aceclofenac/pantoprazole loaded zein/eudragit S 100 nanofibers were developed using a single nozzle electrospinning process to obtain the sustained release of both the drugs up to 8 h to reduce the gastrointestinal side-effect of aceclofenac [28]. In contrast to the combined formulation, our concept was to enhance the bioavailability and reduce the gastrointestinal side effect of aceclofenac with orally dissolving nanofibrous webs, which can provide fast buccal absorption of active. The present study aimed to formulate and characterize aceclofenac-loaded nanofiber-based orally dissolving webs from triethanolamine-containing polyvinylpyrrolidone (PVP) by electrospinning. The morphology, thermal properties, drug content and drug release profile of the ternary systems were analyzed in order to select the right polymer and active substance concentrations concerning the properties of potential orally dissolving webs.

## 2. Materials and Methods

### 2.1. Materials

**Figure 1:**
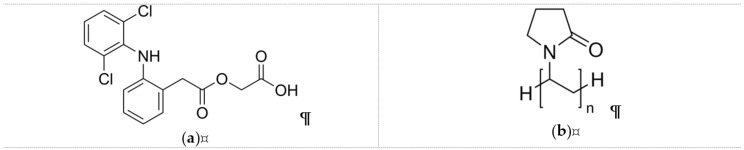
Chemical structures of (**a**) aceclofenac and (**b**) polyvinylpyrrolidone (PVP).

The active pharmaceutical, aceclofenac (Figure 1a) was provided by Richter Gedeon Plc. (Budapest, Hungary). For the preparation of the viscous polymeric solutions, poly(vinyl-pyrrolidone) (PVP K90, Kollidon K90, Figure 1b) was used, which was obtained from Sigma Aldrich (Merck, Darmstadt, Germany). Triethanolamine (trolamine) and ethanol were from Molar Chemicals (Budapest, Hungary). Ultrapure, distilled water was prepared in-house by a MilliQ water purification system. Potassium dihydrogen phosphate and disodium hydrogen phosphate (Merck KGaA, Darmstadt, Germany) were employed for the preparation of the phosphate buffer solution (1 M, pH 6.8) used for the in vitro dissolution study.

### 2.2. Methods

#### 2.2.1. Preparation of PVP Solutions Containing Aceclofenac

The active substance was dissolved in a solvent mixture consisting of ethanol and distilled water in a ratio of 75:25 (*w*/*w*), obtaining a final concentration of 10 mg/g. Trolamine and PVP was dissolved in the obtained solution (amounts described in Table 1) under magnetic stirring until a clear gel was obtained (700 rpm, 1 h with an Ikamag RET magnetic stirrer).

#### 2.2.2. Electrospinning Process

The resulting gel was transferred into a 1 mL plastic syringe equipped with a metallic needle (22G–0.7 mm inner diameter). The needle to the collector distance was 10, 12.5 and 15 cm, while the applied voltage was examined at 14, 15 and 16 kV. For the final formulation, the distance between the spinneret and the collector was set to 12.5 cm and the applied voltage to 15 kV, the flow rate was set at 0.1 µL/s. The nanofibers were obtained using the eSpin Cube device (SpinSplit Ltd., Budapest, Hungary), equipped with an NT-35 high voltage DC supply (Unitronik Ltd., Nagykanizsa, Hungary) and collected on a vertically-positioned, grounded aluminum plate covered with parchment paper.

#### 2.2.3. Scanning Electron Microscopic Analysis (SEM)

The morphology of the nanofibers was investigated with a scanning electron microscope (SEM). The samples were fixed by a double-sided carbon adhesive tape and then coated by a sputtered gold conductive layer (JEOL JFC-1200 Fine Coater, JEOL Ltd., Tokyo, Japan). SEM images were taken with a JEOL JSM-6380LA scanning electron microscope (JEOL Ltd., Tokyo, Japan) at 500×, 1500× and 5000× magnifications.

The diameters of the fibers were measured with the aid of the ImageJ software (US National Institutes of Health, Bethesda, MD, USA) and the average fiber diameter was calculated based on 50 different randomly selected individual filaments at 5000× magnification.

#### 2.2.4. Fourier-Transform Infrared (FT-IR) Spectroscopy

Physicochemical properties of the nanofibers were studied using the Jasco FT/IR-4200 spectrophotometer equipped with the Jasco ATR PRO470-H single reflection accessory (Jasco Corporation, Tokyo, Japan). The measurements were accomplished in the absorbance mode, and spectra were collected over a wavenumber range of 4000 and 400 cm^−1^. 100 scans were performed at a resolution of 4 cm^−1^.

#### 2.2.5. Differential Scanning Calorimetry (DSC)

Thermograms of aceclofenac, PVP, physical mixture and nanofibers with and without the drug were recorded on a Shimadzu DSC-60 type of thermal analyzer (Shimadzu Corporation, Kyoto, Japan). Samples were accurately weighted in aluminium pans, sealed and scanned from 27 to 250 °C under air atmosphere with a rate of 10 °C/min. Al_2_O_3_ was used as reference.

#### 2.2.6. Determination of Drug Content of Aceclofenac-Loaded Nanofibers

The absorbance of samples was measured on 277 nm absorption band of aceclofenac using the Shimadzu UV-1601PC UV spectrophotometer (Shimadzu Corporation, Kyoto, Japan). The stock solution was prepared by dissolving 10 mg aceclofenac in 10 mL ethanol, then diluting 1 mL to 50 mL with distilled water, resulting in a final concentration of 20 µg/mL. The drug content was measured by following the absorbance of the solution prepared by dissolving the nanofibers in the same way. Samples were assayed from three consecutive electrospinning runs (*n* = 3).

#### 2.2.7. In Vitro Drug Dissolution Test

Small volume dissolution tests were performed in an in-house assembled dissolution setup, as described in our earlier publication [29]. In order to demonstrate the small-volume dissolution in the oral cavity, dissolution tests of aceclofenac-loaded nanofibers were investigated in 20 mL of dissolution medium, at 37.0 ± 1 °C. As dissolution medium phosphate buffer (pH 6.8, 1 M, Ph. Eur. 8) was prepared. Aceclofenac samples (around 3 mg) and N2 fiber samples (corresponding to 3 mg aceclofenac, around 50 mg nanofiber, each) were put into six test tubes equipped with a magnetic stirring bar. The stir rate was set to 200 rpm. The dissolution was followed for 30 min. At predetermined time points (1 min, 3 min, 5 min, 10 min, 15 min, 30 min), 1 mL samples were withdrawn and filtered through a 10 µm Whatman filter. 300 μL sample was diluted to 3.0 mL with dissolution media. Aceclofenac concentration was determined spectrophotometrically at 277 nm.

## 3. Results and Discussion

### 3.1. Morphology of the Aceclofenac-Loaded Nanofibers

**Figure 2:**
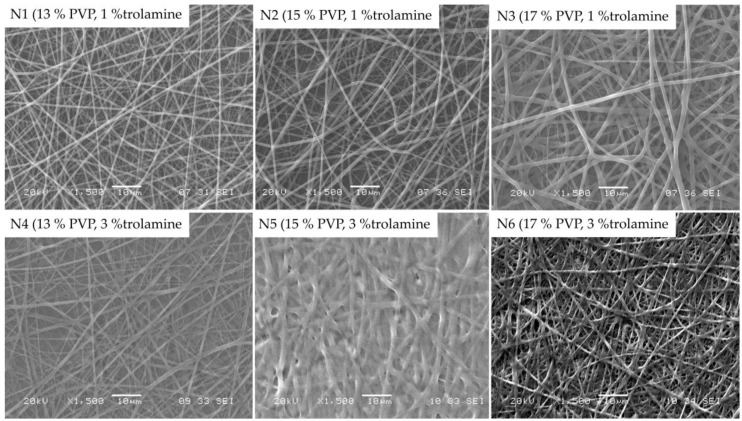
SEM morphology of different samples at 1500× magnification.

Morphology of the obtained nanofibers were examined by SEM. Figure 2 represents the SEM images of samples with 13%, 15% and 17% PVP content with different trolamine proportions, with 12.5 cm emitter to collector distance.

When compared to the 1% trolamine content, on the images of samples with 3% trolamine, the deterioration of nanofibers can be observed, which could be the consequence of the plasticization effect of higher trolamine content. The latter is in good agreement with the results of the authors’ previous works [29,30], where the film formation occurred as a result of the glassy-to-rubbery transition of the polymeric carriers. The moisture absorbed from the environment acts also as a plasticizer leading to a significant decrease of the *T*_g_ of the fiber-forming polymers. Among the samples with 1% trolamine content, the fibers with 15% PVP content proved to be the most suitable for their intended use. The obtained fibers presented diameters from approximately 200 to 800 nanometers with an average of 596 ± 215 nm, with smooth, uniform surfaces. Thus, the composition N2 was used for further studies. Along with the increase of the trolamine concentration the nanofiber structure was shifted to film formation, depending on the polymer-trolamine ratio of the composite fibers. This phenomenon was visualized on the SEM photos (Figure 2, 3% trolamine-containing samples) and can be explained by the enhanced molecular mobility of the plasticized fibers and thus the decreased inner cohesion within the polymeric chains.

### 3.2. FT-IR Analysis

**Figure 3:**
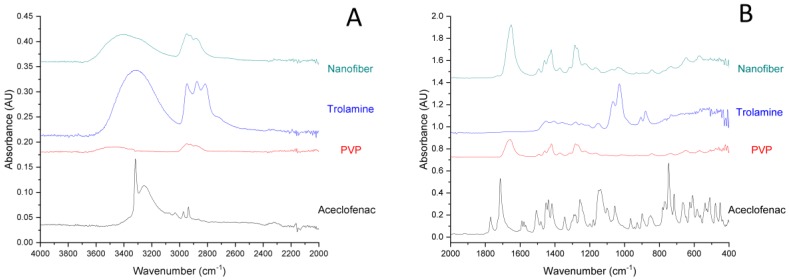
Expanded FT-IR spectra (**A**) 4000–2000 cm^−1^; (**B**) 2000–400 cm^−1^) of individual components and the nanofibrous formulation (composition N2: 10 mg/g aceclofenac, 15 *w*/*w*% PVP, 1 *w*/*w*% trolamine in ethanol:water 75:25 *w*/*w*%).

FT-IR spectra of the components and aceclofenac-loaded nanofibers are represented in Figure 3. In accordance with earlier reports, the IR spectrum of pure aceclofenac shows characteristic peaks at around 3318 cm^−1^ (Figure 3A), which can be attributed to the secondary amine N-H stretching or carboxylic acid band (O-H stretching), two ketone bands at around 1714 and 1770 cm^−1^ and multiple phenyl ring bands in the fingerprint region (Figure 3B) [31]. In the case of the fiber-forming polymer, PVP presents a strong absorption peak at 1650 cm^−1^, characteristic for the carbonyl group (C=O stretching). The band at 1419 cm^−1^ is the –CH_2_ bending vibration, while the band at 1285 cm^−1^ is usually assigned to the C-N stretching vibrations [32]. The IR spectrum of trolamine displays a broad absorption peak at around 3330 cm^−1^, which corresponds to O-H stretching, while those at 2817, 2874 and 2947 cm^−1^, respectively are assigned to fundamental vibrations of the CH bond. IR spectrum of the nanofibrous sample displays the abovementioned characteristic bonds of both PVP and trolamine, however, none of the absorption peaks of aceclofenac is clearly distinguishable. The latter could refer to that the dissolved initially crystalline aceclofenac remained amorphous in the nanofibrous formulation. On the other hand, in the region of 1500–500 cm^−1^ (fingerprint region) the characteristic peaks of trolamine can be identified (882, 909, 1032 and 1071 cm^−1^, respectively).

### 3.3. DSC Measurements

**Figure 4:**
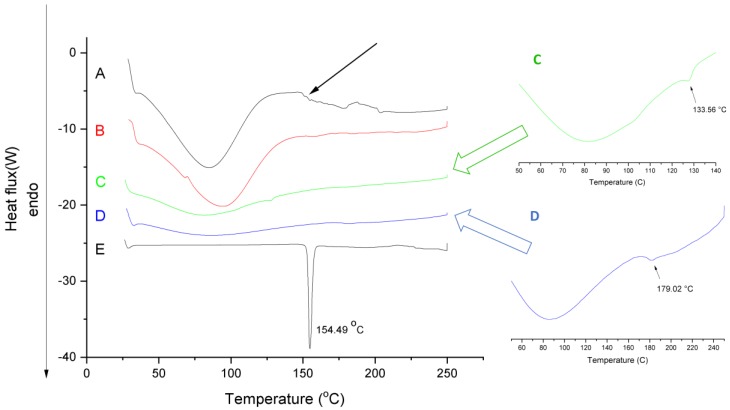
Differential scanning calorimetry (DSC)thermograms of the (A) nanofibrous formulation (composition N2 of polymeric solution: 10 mg/g aceclofenac, 15 *w*/*w*% PVP, 1 *w*/*w*% trolamine in ethanol:water 75:25 *w*/*w*%); (B) neat fiber; (C) physical mixture (same amount of PVP and aceclofenac as in composition N2); (D) PVP and (E) aceclofenac.

Figure 4 shows the DSC curves of aceclofenac, PVP, physical mixture, neat fiber and aceclofenac-loaded nanofiber.

The thermogram of aceclofenac presents a sharp endotherm peak at 154.49 °C, indicating its melting point. The melting-point depression of aceclofenac (approximately 133.6 °C) can be observed in the physical mixture (see green insert) showing the effect of the adsorption of PVP and trolamine on the surface of aceclofenac and consequently modifying its thermal properties. The small peak intensity of the aceclofenac melting endotherm refers to its proportion in the physical mixture. On the curves of aceclofenac-loaded nanofibers, there is a lack of melting endotherm, probably due to the amorphization of aceclofenac. Broad endothermic peaks below 100 °C on the thermograms of neat, placebo fibers and aceclofenac-loaded fibers can be associated with the moisture content of the prepared samples. The increased moisture content of the fibrous samples is associated with their larger surface area compared to the powder substances; thus, they are prone to absorb more water. Based on the extent of the peaks, it can be concluded, that the increased hygroscopicity of the fibers, on the one hand, can have a significant impact on the dissolution improvement of the active, but on the other hand it could initiate a glassy-to-rubbery transition of the PVP, thus destroying the fibrous structure. Similar pseudopolymorphism of the fiber-base polymer composite can be seen in the authors’ previous stability studies of papaverine hydrochloride-loaded electrospun nanofibrous buccal sheets [30]. The glass transition temperature of PVP K90 denoted by an endothermic change in the baseline thermal profiles (see blue insert) of reversible heat flow was 179 °C [33], which can be more sensitively detected at 5 °C/min heating rate. The presence of solvent residues (e.g., water) and other excipients, like the solubilizing agent trolamine, decreased the glass transition temperature (indicated with black arrow) and thus modifying the glassy-to-rubbery transition of the fibrous polymeric carriers. The estimated glass transition temperature of ternary fibers (*T*_gmix_) calculated based on the Fox equation (Equation (1), [34]) are as follows

1/*T*_gmix_ = (*w*_1_/*T*_g1_) +( *w*_2_/*T*_g2_) (1)

where *w* = weight fraction of components, *T*_g_ = glass transition temperature of the components.

The predicted glass transition temperature of various fibers, calculated by Equation (1), together with the average diameters, are summarized in Table 1. The presence of low molecular weight additive, trolamine lowers the *T*_g_ of the ternary composite fibers and consequently increases their free volumes. The average diameters of the different composite nanofibers (see Table 1) indicated that along with the increase of the polymer concentration, the obtained fiber diameter also increased at a given plasticizer content of the initial polymer solution. The increased free volume of the fibers results in enhanced water absorption, which enables further lowering of the *T*_gmix_ and showing rubbery properties at lower temperatures by creating ribbon-like fibers.

### 3.4. Determination of Drug Content of Aceclofenac-Loaded Nanofibers

**Figure 5:**
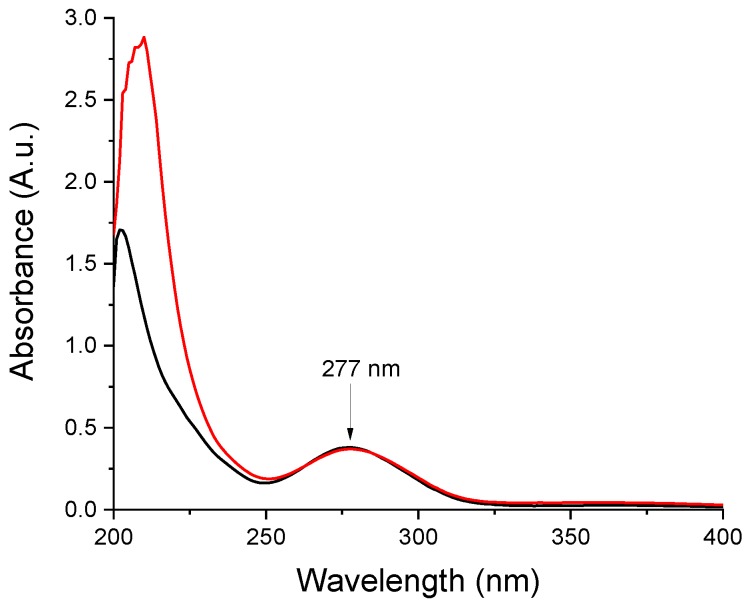
Comparative UV spectra recorded of ethanolic solutions of-red trace-the nanofibrous formulation (composition N2 of polymeric solution: 10 mg/g aceclofenac, 15 *w*/*w*% PVP, 1 *w*/*w*% trolamine in ethanol:water 75:25 *w*/*w*%); black trace–neat aceclofenac.

In order to confirm the presence of the active in the prepared nanofibers, comparative UV spectra were recorded for ethanolic solutions of neat aceclofenac and the nanofibrous web obtained from composition N2 (Figure 5). The obtained spectra are similar, except the low UV region, where the solution obtained from the nanofibrous formulation displays a higher absorption due to the matrix components. For both spectra, λ_max_ values were recorded at 277 nm (i.e., the expected UV maximum for aceclofenac). Based on the UV spectrometric measurements, the nanofibrous webs contain 5.77 ± 0.12 *w*/*w*% aceclofenac, which corresponds to 98.19 ± 2.04% of the theoretical aceclofenac content (5.88 *w*/*w*%)

### 3.5. Dissolution Measurements of the Nanofibers

**Figure 6:**
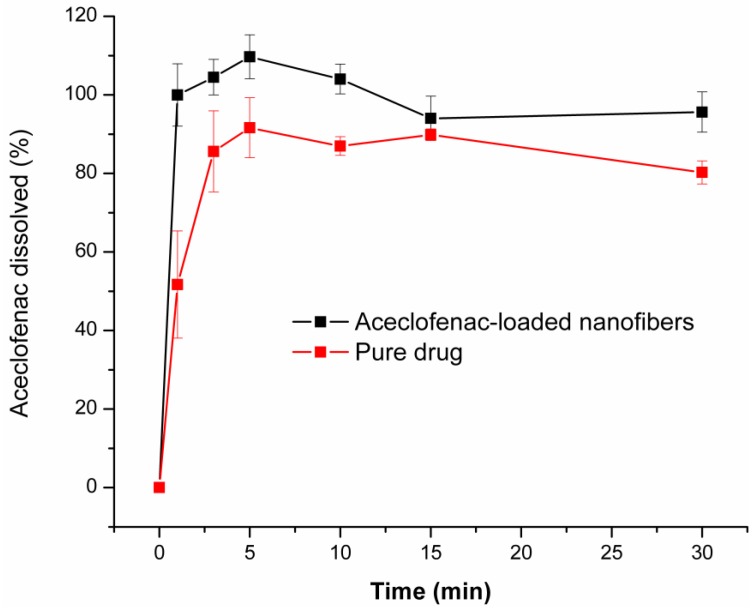
Dissolution profile of aceclofenac-loaded nanofibers (composition N2 of polymeric solution: 10 mg/g aceclofenac, 15 *w*/*w*% PVP, 1 *w*/*w*% trolamine in ethanol:water 75:25 *w*/*w*%) and pure active substance (average of three measurements ± SD).

Figure 6 illustrates the obtained comparative dissolution profiles of the drug-loaded nanofibers (composition N2 of polymeric solution) and neat aceclofenac. As it can be observed, drug release is spontaneous from the nanofibrous webs and complete even at the first minute of the low volume dissolution test. Although, around 90% of the selected dose of the pure active substance dissolves throughout the dissolution test, the dissolution rate is slower in comparison to the nanofibrous formulation (51% and 85% at 1 min and 3 min, respectively). The faster dissolution rate of the fibrous formulation can be explained by the increased specific surface area of the nanofibers, the amorphous state of the active and the highly hydrophilic nature of the fiber-forming polymer.

## 4. Conclusions

This study demonstrated that the nanofiber forming process is influenced by multiple parameters such as the composition of the aceclofenac-containing viscous solution and the electrospinning parameters. The SEM studies confirmed the nanofibrous structure, while the FT-IR and DSC tests can indicate that the originally crystalline aceclofenac was in the amorphous form in the nanofibrous formulations. Upon exposure to aqueous media, dissolution is believed to generate a supersaturated state due to the amorphous state of the drug. The dissolution of the polymer matrix was fast and complete. The selected composition of the triethanolamine-aceclofenac-poly(vinyl-pyrrolidone) ternary composite resulted in an amorphous drug-loaded electrospun nanofibrous structure of fast and complete dissolution in aqueous media. Based on the results the formulated aceclofenac-loaded orally dissolving webs provide a promising alternative in the therapy of osteoarthritis and rheumatoid arthritis with an enhanced rate and extent of absorption due to the improved wettability and dissolution rate thus providing smaller effective doses and causing fewer potential side effects.

## Figures and Tables

**Table 1:** Composition of viscous polymeric solutions used for the preparation of nanofibers, diameters of the obtained fibers and estimation of the glass transition temperatures.

Sample No.	Aceclofenac Concentration (mg/g)	PVP Concentration (*w*/*w*%)	Trolamine Concentration (*w*/*w*%)	Diameters (nm)	Predicted *T*_gmix_ (°C)
N1	10	13	1	435 ± 159	139.7
N2	10	15	1	596 ± 215	143.6
N3	10	17	1	1046 ± 113	147.1
N4	10	13	3	757 ± 157	89.9
N5	10	15	3	786 ± 322 *	97.9
N6	10	17	3	812 ± 244 *	104.5

* The average diameters were determined from the fibrous elements of the sample, bias.
